# Supplementary material for: Multi-Compartment, Early Disruption of cGMP and cAMP Signalling in Cardiac Myocytes from the mdx Model of Duchenne Muscular Dystrophy
Source: Int J Mol Sci. 2020 Sep 25;21(19):7056. doi: 10.3390/ijms21197056 (PMC7582831; doi:10.3390/ijms21197056)
Supplement: Supplementary file 1 [file ijms-21-07056-s001.pdf]

Supplementary Information

Supplementary Figure 1

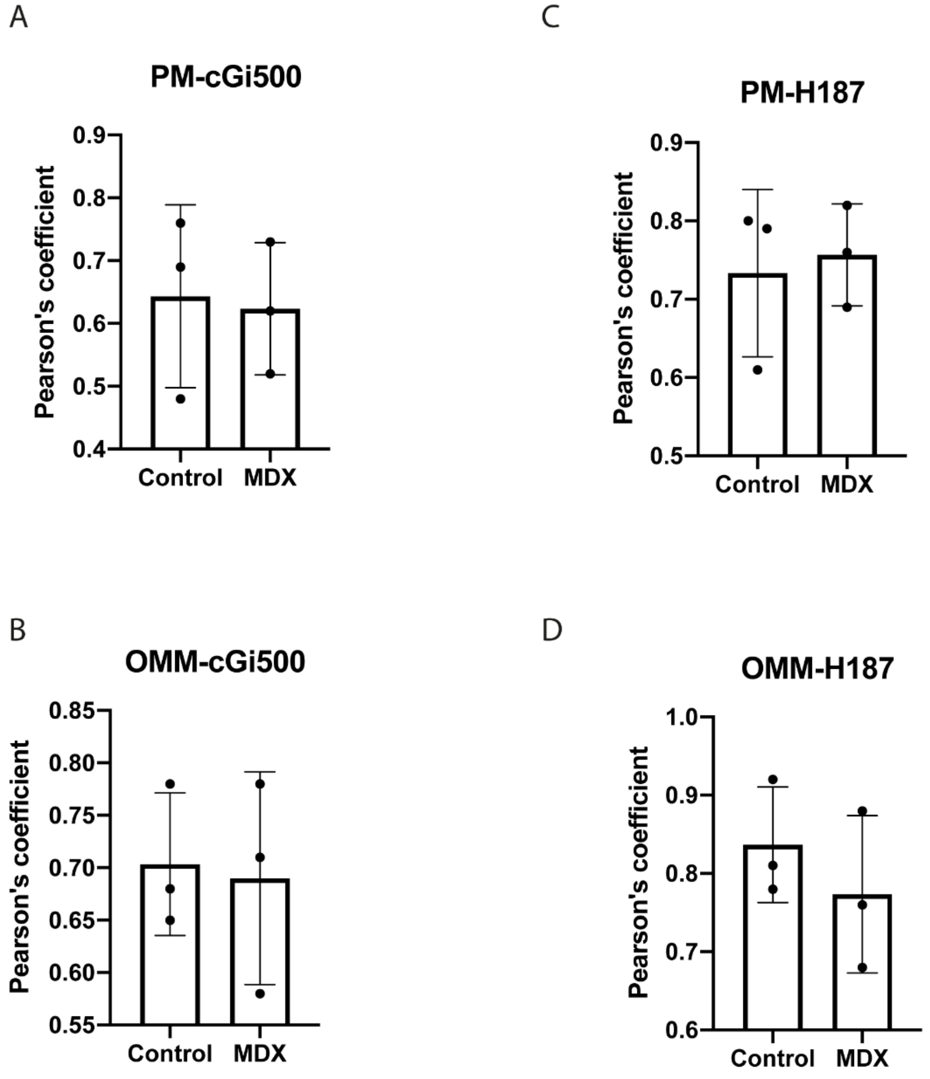

Figure S1. Pearson's correlation coefficient calculated from images of NVM from control or *mdx* mice expressing PM-cGi500 (**A**) or PM-H187 (**B**), co-stained with the plasma membrane marker WGA, or expressing OMM-cGi500 (**C**) or OMM-H187 (**D**), labelled with the mitochondrial dye mitotracker. Values are mean ± SEM. N = 3 biological replicates. Student's T-test shows no statistically significant difference.

Supplementary Figure 2

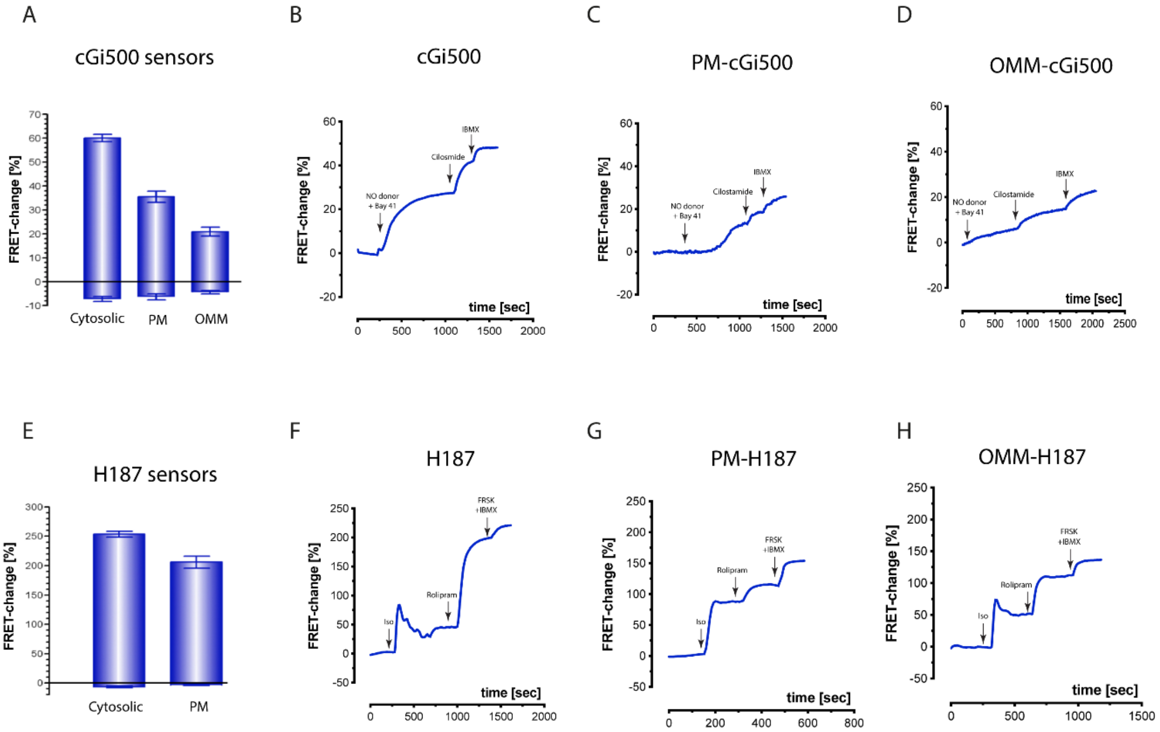

Figure S2. (**A**) FRET change recorded for the cytosolic and targeted cGi500 reporters measured in CHO cells upon saponin permeabilization and bath application of a 1mM cGMP solution (columns top) or a solution containing no cGMP (columns bottom). Taken together, these positive and negative changes represent the dynamic range of the respective sensor. (B,C,D): Representative kinetics of the time course response to sGC activation and PDE3 inhibition recorded in NVM from control mice expressing the cytosolic cGi500 (**B**), the plasmalemma targeted PM-cGi500 (**C**) and the OMM-cGi500 targeted to the outer mitochondrial membrane (**D**). (**E**) Dynamic range for the cytosolic and plasmalemma targeted reporter H187 measured in CHO cells upon micro-infusion of 1mM cAMP or zero cAMP. The dynamic range for OMM-H187 was previously shown not to differ from that of cytosolic H187 [1]. (F, G, H): Representative kinetics of the time-course response to β-AR activation and PDE4 inhibition recorded in NVM from control mice expressing cytosolic H187 (**F**), the plasmalemma targeted PM-H187 (**G**) and the OMM-H187 targeted to the outer mitochondrial membrane (**H**). In all cases, at the end of the experiment 100 μM IBMX or a combination of 100 μM IBMX and 25 μM was applied to check for saturation of the sensor. Values for (A) and (E) are mean ± SEM, n ≥ 3 for each condition.

Supplementary Figure 3

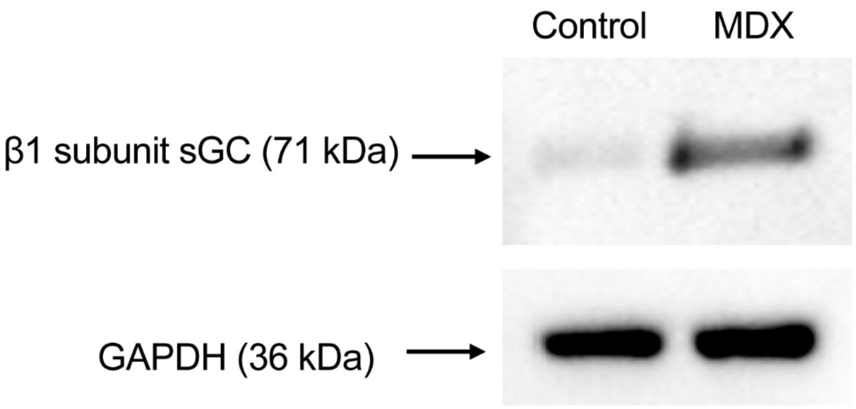

Figure S3. Expression level of sGC subunit β1 as determined by western blot analysis of NVM whole cell lysates obtained from control or *mdx* mice. Shown blots are representative for two independent experiments.

Supplementary Figure 4

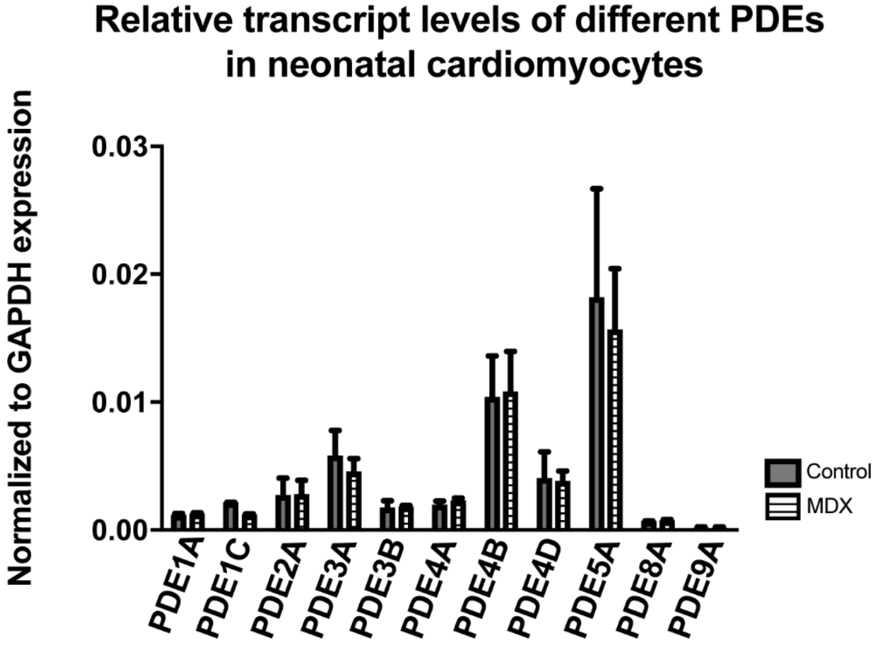

Figure S4. Comparison of cAMP- and cGMP-PDE mRNA quantification in NVM from control and *mdx* mice. Values are mean ± SEM. N = 3 biological replicates. For all PDE isoforms Student's *t*-test shows no statistically significant difference.

Supplementary Figure 5

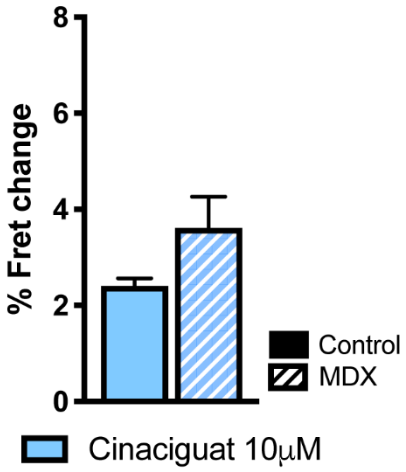

Figure S5. cGMP response elicited in the cytosol of NVM from control or *mdx* mice expressing the cGi500 sensor. Values are mean ± SEM. N = 3 biological replicates. Student's T-test shows no statistically significant difference.

Supplementary Figure 6

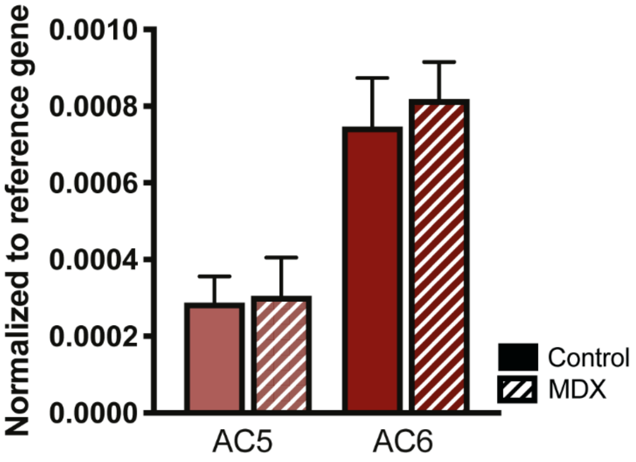

Figure S6. AC5 and AC6 mRNA quantification in NVM from control and *mdx* mice. Values are mean ± SEM. N=3 biological replicates. Student's *t*-test shows no statistically significant difference.

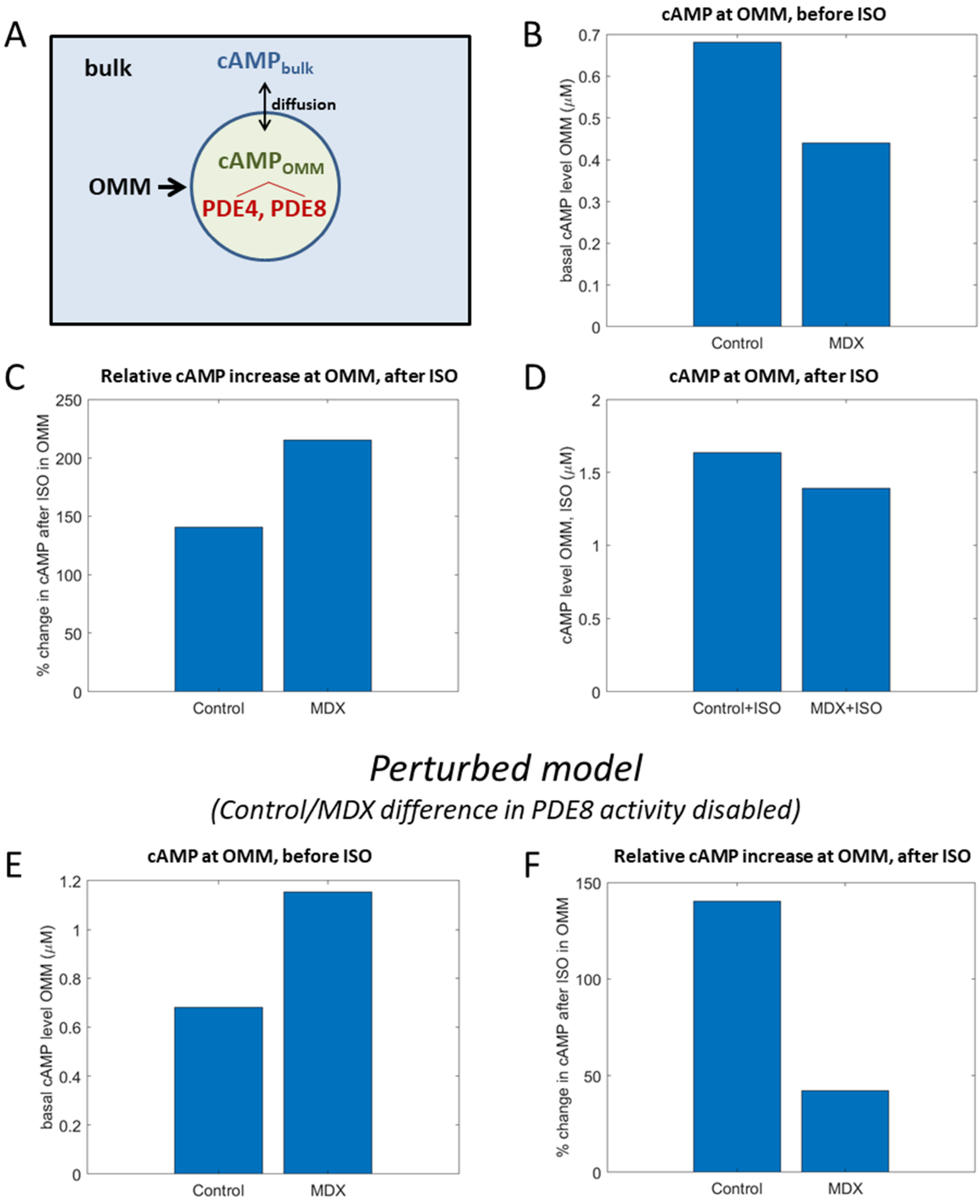

Figure S7. Simulation of PDE activity at the OMM. (**A**) A two-compartment model of local cAMP regulation at OMM. (**B**) Simulated basal cAMP concentration at the OMM for control and *mdx* cardiac myocytes. (**C**) Simulated percent change in cAMP at OMM following ISO exposure. (**D**) Simulated cAMP concentration at the OMM following ISO exposure. (**E, F**) An analogy of panels B, C for a model where *mdx* PDE8 concentration is set to be the same as in control.

To complement experimental observations in the study, we carried out additional simulations of simple local control of cAMP at the OMM. The model represents cAMP levels in the bulk cytosol as well as at OMM, where cAMP is hydrolysed by PDE4 and PDE8 (Figure S7A, see below for simulation details). Simulated levels of the PDEs are different for control and *mdx* cardiac myocytes (PDE4 being slightly lower in *mdx*, and PDE8 being substantially increased). The model predicts that cAMP levels are lower in *mdx* than in controls at the OMM (Figure S7B), consistent with the experimental results (Figure 6B). This is observed despite the fact that the simulated cAMP levels in bulk cytosol are substantially higher in *mdx* compared to control cells, consistent with Figure 4B. Simulated ISO stimulation (represented as increased bulk cAMP, and substantial phosphorylation of PDE4 and PDE8 in OMM) shows a greater relative increase in cAMP at the OMM (Figure S7C), consistent with experimental data (Figure 6D). At the same time, the increased response to ISO at the OMM of *mdx* cells does not imply that the absolute concentration of cAMP is increased; on the contrary, the model suggests that the concentration of cAMP at OMM remains diminished in *mdx* cells compared to controls (Figure S7D). The greater response shown in Figure S7C therefore results simply from the fact that the relative difference between cAMP levels of controls and *mdx* is smaller after ISO (Figure S7D) than in basal conditions (Figure S7B).

Further investigation reveals that the good agreement of simulations above with experimental data strongly depends on the representation of the fact that PDE8 is markedly elevated in *mdx* versus control, reducing the OMM cAMP. When the model is simulated with *mdx* having the same PDE8 concentration as controls, data in Figure 6B are not matched anymore (Figure S7E) as opposed to the original model (Figure S8B), and neither are the data in Figure 6D (new model shown in Figure S7F, as opposed to the original one in Figure S7C). Together, our simulations suggest a key role of PDE8 in regulation of cAMP levels at the OMM.

Simulation details

The general approach to model construction is based on the comprehensive model of local control of β-adrenergic stimulation by Heijman et al. [1] but our model is much simpler, consisting of only two compartments: bulk cytosol and OMM (Figure S7A). The model has multiple parameters which allow representation of distinct properties of control and *mdx* cells, as well as representation of ISO stimulation.

Bulk cytosol is represented as a single constant value of $cAMP_{bulk}$ representing cAMP concentration in μM. $cAMP_{bulk}$ = 1 μM for control cells based on [2], and is increased to 1.5 μM for *mdx* cells, based on Figure 4B. Following simulated ISO stimulation, controls $cAMP_{bulk}$ = 10 μM, and *mdx* $cAMP_{bulk}$ = 8 μM, estimated based on the calibration curve for the H187 sensor [3].

OMM represents a dynamically evolving concentration of cAMP ($cAMP_{OMM}$), which depends on the diffusion between bulk and OMM, as well as the activity of PDEs at the OMM (which may or may not be phosphorylated and activated, depending on the presence of ISO). Properties of PDEs are determined by affinity for cAMP K_m, and the turnover number K_{cat}. For PDE4, $K_{m,PDE4}$ = 5 μM [4] $K_{cat,PDE4}$ = 0.045 s^{-1} [4], and $K_{cat,PDE4,phosphorylated}$ = 2.3 s^{-1} [3]. For PDE8, $K_{m,PDE8}$ = 0.06 μM [5], $K_{cat,PDE8}$ = 0.225 s^{-1} [3] and $K_{cat,PDE8,phosphorylated}$ = 0.27 s^{-1}. I.e., full phosphorylation increases the activity of PDE4 50 fold, and the activity of PDE8 by 20% [6]. In the presence of ISO, a 70% phosphorylation of PDEs is assumed, based on the level of stimulated cAMP and cAMP concentration dependency of PKA activity [3].

Based on [3], the concentration of PDE4 at the OMM for controls is $[PDE4]_{OMM,ctr}$ = 0.12 μM, which is slightly reduced in *mdx* ($[PDE4]_{OMM,mdx}$ = 0.09 μM).

Concentration of PDE8 in OMM for WT is $[PDE8]_{OMM,ctr}$ = 0.006 μM, estimated as 5% of the PDE4 concentration, based on [3]. According to Fig 6C, OMM PDE8 was substantially increased for *mdx* cells: $[PDE8]_{OMM,mdx}$ = 0.03 μM.

In absence of OMM-specific measurements, the diffusion flux rate ($J_{bulk/OMM} = 0.9 \cdot 10^{-8}$ μLs^{-1}) and OMM volume ($V_{OMM} = 1.5204 \cdot 10^{-6}$ μL) were set as the extracaveolar-cytosolic diffusion rate, and the extracaveolar volume in the Heijman model [1].

The equation for cAMP hydrolysis by PDE4 is:

$$\frac{dCAMP_{PDE4}}{dt} = \frac{(1-\theta_p)\cdot K_{cat,PDE4}\cdot [PDE4]_{OMM}\cdot cAMP_{OMM} + \theta_p \cdot K_{cat,PDE4,phosphorylated}\cdot [PDE4]_{OMM}\cdot cAMP_{OMM}}{K_{m,PDE4} + cAMP_{OMM}},$$

where θ_p is the fraction of phosphorylated PDE4 (0 for control condition and 0.7 for ISO stimulation).

The equation for cAMP hydrolysis by PDE8 is analogical:

$$\frac{dCAMP_{PDE8}}{dt} = \frac{(1-\theta_p)\cdot K_{cat,PDE8}\cdot [PDE8]_{OMM}\cdot cAMP_{OMM} + \theta_p \cdot K_{cat,PDE8,phosphorylated}\cdot [PDE8]_{OMM}\cdot cAMP_{OMM}}{K_{m,PDE8} + cAMP_{OMM}}$$

Diffusion of cAMP from bulk to OMM is as follows:

$$diffusion = J_{bulk/OMM}\frac{cAMP_{bulk} - cAMP_{OMM}}{V_{OMM}}$$

The equation for derivative of $cAMP_{OMM}$ is:

$$\frac{dCAMP_{OMM}}{dt} = -\left(\frac{dCAMP_{PDE4}}{dt} + \frac{dCAMP_{PDE8}}{dt}\right) + diffusion$$

References.

1. Burdyga, A.; Surdo, N.C.; Monterisi, S.; Di Benedetto, G.; Grisan, F.; Penna, E.; Pellegrini, L.; Zaccolo, M.; Bortolozzi, M.; Swietach, P. Phosphatases control PKA-dependent functional microdomains at the outer mitochondrial membrane. *Proc. Natl. Acad. Sci.* **2018**, *115*, E6497–E6506.
2. Heijman, J.; Volders, P.G.A.; Westra, R.L.; Rudy, Y. Local control of β-adrenergic stimulation: Effects on ventricular myocyte electrophysiology and Ca2+-transient. *J. Mol. Cell. Cardiol.* **2011**, *50*, 863–871.
3. Surdo, N.C.; Berrera, M.; Koschinski, A.; Brescia, M.; Machado, M.R.; Carr, C.; Wright, P.; Gorelik, J.; Morotti, S.; Grandi, E. FRET biosensor uncovers cAMP nano-domains at β-adrenergic targets that dictate precise tuning of cardiac contractility. *Nat. Commun.* **2017**, *8*, 1–14.
4. Koschinski, A.; Zaccolo, M. Activation of PKA in cell requires higher concentration of cAMP than in vitro: Implications for compartmentalization of cAMP signalling. *Sci. Rep.* **2017**, *7*, 1–12.
5. Bender, A.T.; Beavo, J.A. Cyclic nucleotide phosphodiesterases: Molecular regulation to clinical use. *Pharmacol. Rev.* **2006**, *58*, 488–520.
6. Fisher, D.A.; Smith, J.F.; Pillar, J.S.; Denis, S.H.S.; Cheng, J.B. Isolation and characterization of PDE8A, a novel human cAMP-specific phosphodiesterase. *Biochem. Biophys. Res. Commun.* **1998**, *246*, 570–577.
7. Brown, K.M.; Lee, L.C.Y.; Findlay, J.E.; Day, J.P.; Baillie, G.S. Cyclic AMP-specific phosphodiesterase, PDE8A1, is activated by protein kinase A-mediated phosphorylation. *Febs Lett.* **2012**, *586*, 1631–1637.

.
